# TREM2 in CNS homeostasis and neurodegenerative disease

**DOI:** 10.1186/s13024-015-0040-9

**Published:** 2015-09-04

**Authors:** Meghan M. Painter, Yuka Atagi, Chia-Chen Liu, Rosa Rademakers, Huaxi Xu, John D. Fryer, Guojun Bu

**Affiliations:** Department of Neuroscience, Mayo Clinic, 4500 San Pablo Road, Jacksonville, FL 32224 USA; Fujian Provincial Key Laboratory of Neurodegenerative Disease and Aging Research, Institute of Neuroscience, College of Medicine, Xiamen University, Xiamen, Fujian 361102 China; Neurobiology of Disease Graduate Program, Mayo Clinic College of Medicine, 4500 San Pablo Road, Jacksonville, FL 32224 USA

**Keywords:** Alzheimer’s disease, TREM2, Microglia, Inflammation

## Abstract

Myeloid-lineage cells accomplish a myriad of homeostatic tasks including the recognition of pathogens, regulation of the inflammatory milieu, and mediation of tissue repair and regeneration. The innate immune receptor and its adaptor protein—triggering receptor expressed on myeloid cells 2 (TREM2) and DNAX-activating protein of 12 kDa (DAP12)—possess the ability to modulate critical cellular functions via crosstalk with diverse signaling pathways. As such, mutations in TREM2 and DAP12 have been found to be associated with a range of disease phenotypes. In particular, mutations in TREM2 increase the risk for Alzheimer’s disease and other neurodegenerative disorders. The leading hypothesis is that microglia, the resident immune cells of the central nervous system, are the major myeloid cells affected by dysregulated TREM2-DAP12 function. Here, we review how impaired signaling by the TREM2-DAP12 pathway leads to altered immune responses in phagocytosis, cytokine production, and microglial proliferation and survival, thus contributing to disease pathogenesis.

## Background

Host survival is dependent on the detection and clearance of diverse virulent pathogens by the innate and adaptive immune systems. Cells of the myeloid lineage, including macrophages and dendritic cells (DCs), play a pivotal role in both sectors of immunity—they are vital in direct microbe sensing and localized attenuation, and are also responsible for recruitment of other myeloid and lympohocyte effector populations. Interestingly, host receptors normally associated with microbial detection are also able to respond to acute tissue injury through recognition of self-derived damage-associated motifs. How innate immune cells balance the timing and composition of their cytokine cocktails is critical for efficient clearance of pathogen and cellular debris without excessive inflammation and tissue destruction. Genetic association studies have demonstrated that dysregulated innate immune signaling is linked to numerous neurological and immunological disease phenotypes [1–7]. Recently, mutations in the innate immune receptor-adaptor duo, triggering receptor expressed on myeloid cells 2 (TREM2) and DNAX-activating protein of 12 kDa (DAP12), have been reported in a spectrum of neurodegenerative disorders including Nasu-Hakola disease (NHD; or polycystic lipomembranous osteodysplasia with sclerosing leukoencephalopathy, PLOSL) [8–11], frontotemporal dementia (FTD) [12–17], Alzheimer’s disease (AD) [13, 17–23], Parkinson’s disease (PD) [14], amyotrophic lateral sclerosis (ALS) [24], and essential tremor [25]. Three non-exclusive hypotheses suggest roles for TREM2-DAP12 signaling during homeostasis and how mutations may contribute to altered neuro-immunological function: (1) TREM2-DAP12 suppresses inflammatory cytokine production following receptor-mediated recognition of microbial or self-derived damage-associated motifs; (2) TREM2-DAP12 mediates phagocytosis of a variety of microbial and endogenous ligands to facilitate debris clearance following injury or insult; and (3) TREM2-DAP12 signaling induces a transcriptional profile reflective of enhanced myeloid cell proliferation and reduced cell death. In this Review, we discuss the historical evidence of the TREM2-DAP12 genetic link to neurodegenerative diseases and highlight recent data that support a multi-faceted role for microglial TREM2-DAP12 signaling in homeostasis and under disease challenges in the central nervous system (CNS).

## Discovery and validation of *TREM2* as a risk gene for AD and other neurodegenerative diseases

TREM2 belongs to the immunoglobulin (Ig) superfamily of receptors. It contains an extracellular domain, a transmembrane domain, and a short cytoplasmic tail [26]. Oppositely charged intramembrane residues of TREM2 and DAP12 facilitate their interaction [27]. DAP12 is also a type I transmembrane protein with a cytoplasmic tail that harbors a single immunoreceptor tyrosine activation motif (ITAM) [28]. In the periphery, macrophages, immature DCs, and osteoclasts are the primary TREM2-expressing cells *in vivo* [26, 29–31]. In the CNS, TREM2 is highly expressed in microglia with approximately 300-fold higher expression compared to neurons and other glia [32, 33]. DAP12 expression has been reported in cells of both myeloid and lymphoid origin [28, 31]. Although DAP12 is able to interact with greater than twenty other receptors [34], the TREM2-DAP12 axis has received growing attention due to recent genetic association with neurodegenerative diseases [8–25].

Loss-of-function mutations in both TREM2 and DAP12 have been reported to cause NHD/PLOSL, an autosomal recessive disorder in which patients suffer from systemic bone cysts and progressive encephalopathy resulting in presenile dementia [35, 36]. Even though cases of NHD/PLOSL have been described since the 1960s, it wasn’t until the late 1990s that inheritance of the disease was linked to chromosomal region 19q13 [8], and subsequent analysis identified a large chromosomal deletion (exons 1–4) of *TYROBP*, the gene encoding DAP12 [9]. Later, in patients with genetic exclusion of the 19q13 linkage to disease, it was observed that mutations in TREM2 resulted in an identical NHD/PLOSL phenotype [10]. Following this discovery, numerous mutations in TREM2, mostly occurring in exon 2 (IgV domain), have been linked to other forms of neurodegenerative disease.

FTD encompasses a spectrum of disorders that share pathological features of frontal and temporal lobe atrophy, and depending on the cerebral region most affected, clinical symptoms can include language deficits and altered behavior [37]. In an effort to identify novel genes and risk factors associated with disease, patients with an FTD-like clinical diagnosis underwent whole-exome sequencing; the homozygous TREM2 mutations p.Q33X, p.T66M, and p.Y38C were present in affected individuals but not in healthy controls [12]. Also, a nonsense p.W198X mutation [13], and missense p.S116C [16] and p.R47H [14, 16, 17] mutations have been identified in other FTD cohorts. Similar to the NHD/PLOSL patients that harbor TREM2 variant alleles, these FTD patients exhibited characteristic signs of cognitive impairment. Conversely, they did not show symptoms of bone involvement (e.g., bone cysts). Therefore, it was hypothesized that TREM2 may be a risk allele associated with other forms of dementia that have not previously been screened for TREM2 mutations because of their atypical (non-NHD/PLOSL) clinical presentation [12].

Subsequent studies corroborated this hypothesis, and in 2013, two seminal papers described TREM2 mutations in AD [18, 19]. In the elderly, AD is the most common form of dementia with affected individuals worldwide topping 150 million [38]. Familial, early-onset AD (EOAD; <65 years) accounts for <1 % of total cases [39] and are caused by mutations in the *APP, PSEN1,* or *PSEN2* genes [40–42]. Most AD cases are referred to as late-onset AD (LOAD; >65 years) with the greatest genetic risk factor being the ε4 allele of apolipoprotein E *(APOE)* [43, 44]. Mutations associated with both EOAD and LOAD result in toxic amyloid-β (Aβ) accumulation in the CNS (see below).

In addition to the aforementioned impetus to study TREM2 in other forms of dementia, a genome search meta-analysis identified significant evidence for linkage to LOAD at the 6p21.1-q15 locus (which includes *TREM2*) [45]. Therefore, by whole-exome and whole-genome sequencing of AD patients and unaffected individuals, Guerreiro et al. [18] and Jonsson et al. [19] performed genome-wide association analysis with variants that were predicted to affect protein function. In independent patient cohorts, both research groups found that the TREM2 SNP rs75932628-T, encoding the putative p.R47H variant, conferred a significantly increased risk of LOAD with odds ratios of 5.05 [18] and 2.92 [19]—which are comparable to that of *APOE* ε4 [46]. Numerous studies in different ethnic populations have since confirmed this pivotal finding that the p.R47H variant of TREM2 is associated with LOAD [13, 17, 20–23]. Furthermore, this SNP has been linked to other neurodegenerative disorders including PD [14], ALS [24], and essential tremor [25].

## Neuroinflammation in AD

Although overt clinical symptoms of AD do not typically develop until age 65 years and older, disease-initiating events most likely begin decades before symptoms of cognitive decline [47]. The pathological features of AD include amyloid plaques primarily composed of Aβ and neurofibrillary tangles (NFTs) that are aggregates of hyperphosphorylated microtubule-associated protein tau [48]. The amyloid cascade hypothesis of AD focuses on the process of Aβ accumulation and aggregation, which trigger a wave of secondary pathogenic events that lead to eventual synaptic and neuronal degeneration [39].

Aβ peptides, which are typically 39–43 amino acids in length, are derived from sequential cleavage of amyloid precursor protein (APP) by β- and γ-secretases [49]. During homeostasis, Aβ is continuously generated and removed from brain tissue. However, under pathogenic conditions when Aβ production is elevated or Aβ clearance is impaired, this imbalance leads to accumulation and subsequent formation of toxic oligomers, fibrils, and plaques. Mutations found in patients with familial EOAD—*APP*, or *PSEN1* and *PSEN2*, components of the γ-secretase complex—result in overproduction of amyloidogenic Aβ [50]. Evidence supports impaired Aβ clearance as a major contributing pathway for LOAD [51]. A unifying theme is that Aβ aggregates trigger excessive immune responses which can be harmful to synapses and neurons and further impair Aβ clearance.

Aβ aggregates can activate classical innate immune microbial receptors [48]. For example, during a bacterial infection, the host’s pattern recognition receptor (PRR), toll-like receptor 4 (TLR4), is able to detect bacterial lipopolysaccharide (LPS) as a foreign pathogen-associated molecular pattern (PAMP). In turn, pro-inflammatory pathways are activated which aid in the recruitment of additional myeloid and lymphoid effector populations. It is speculated that through an evolutionarily-conserved mechanism to sense and respond to acute tissue injury, host PRRs are also able to recognize self-derived damage-associated molecular patterns (DAMPs). In this way, aggregated Aβ, acting as a DAMP, can activate pro-inflammatory cytokine production through host PRRs [52], including TLR4 [53]. During infection, such sensor activation induces local inflammation followed by resolution to baseline once pathogen attenuation has occurred. Conversely, accumulation of toxic amyloid causes constitutive PRR-mediated Aβ detection that induces chronic stimulation of the innate immune system [38].

Recently, genome-wide association studies (GWAS) have confirmed an active role for innate immune cells in the regulation of the inflammatory milieu within the CNS. In addition to TREM2, mutations in the myeloid receptor CD33 (also known as Siglec-3) [5–7], complement receptor 1 (CR1) [1], and myeloid cell-expressed membrane-spanning 4-domains subfamily A member 6A (MS4A6A) and MS4A4E [6, 7] have been shown to increase the risk of developing AD. These findings support the hypothesis that aberrant activation or impaired function of the innate immune system contributes to the initiation and propagation of the inflammatory process leading to AD.

## TREM2-DAP12 axis inhibits TLR-induced inflammation

In the CNS, microglia provide protective surveillance for parenchymal cells from both intrinsic and extrinsic insults. As the primary cellular innate immune component, microglia act as the major PAMP/DAMP detectors and are equipped with an assortment of cytosolic, endosomal and plasma-membrane-bound PRRs, including all of the TLR subtypes [54]. Delineating key events in TLR4-mediated induction of pro-inflammatory cytokines has become a major focus of AD research since amyloidogenic proteins have been shown to activate canonical pathogen-associated signal transduction cascades via TLR4 ligation [55–57]. PAMP/DAMP engagement of TLR4 (in complex with MD2 and facilitated by CD14 and LBP) recruits the TIR domain-containing adaptor molecules (including MyD88 and TRIF) which activate NF-κB and mitogen-activated protein kinases (including ERK, JNK, and p38) to induce production of inflammatory cytokines [58].

Similar to TLR4, TREM2 has been described as both a PAMP [59, 60] and a DAMP [61–64] sensor; TREM2 is capable of binding gram-positive and gram-negative bacteria [59, 60] as well as a number of ligands exposed during CNS degeneration including anionic, zwitterionic, and myelin-associated lipids [61, 62], and nucleic acid released from dying cells [64]. Due to overlap in stimulatory ligands, it has been difficult to delineate signals emanating from each receptor pathway; however, TREM2 depletion experiments in combination with TLR4-LPS stimulation have begun to define the exact role each receptor plays in the neuroinflammatory response.

In order to characterize whether TREM2-DAP12 signaling is able to modulate TLR4-mediated inflammatory cytokine production, Turnbull and colleagues generated *Trem2*^*−/−*^ mice [30]. Wild-type (WT) and *Trem2*^*−/−*^ bone-marrow-derived macrophages (BMDMs) and primary peritoneal macrophages were stimulated with LPS and cytokine levels were assayed. In both macrophage populations, pro-inflammatory TNF-α and IL-6 were significantly increased in the absence of TREM2 [30]. Following LPS stimulation, synthesis of pro-inflammatory cytokines were similarly elevated in TREM2-depleted microglia and DCs [62, 63, 65], and in DAP12*-*deficient BMDMs [66]. More recently, knockdown of either TREM2 or DAP12 in the microglial cell line BV2 significantly increased IL-1β and IL-6 following LPS stimulation. Importantly, overexpression of full-length TREM2 or the DAP12-interacting C-terminal fragment (CTF) of TREM2 were able to suppress excessive pro-inflammatory cytokine production [67].

**Fig. 1:**
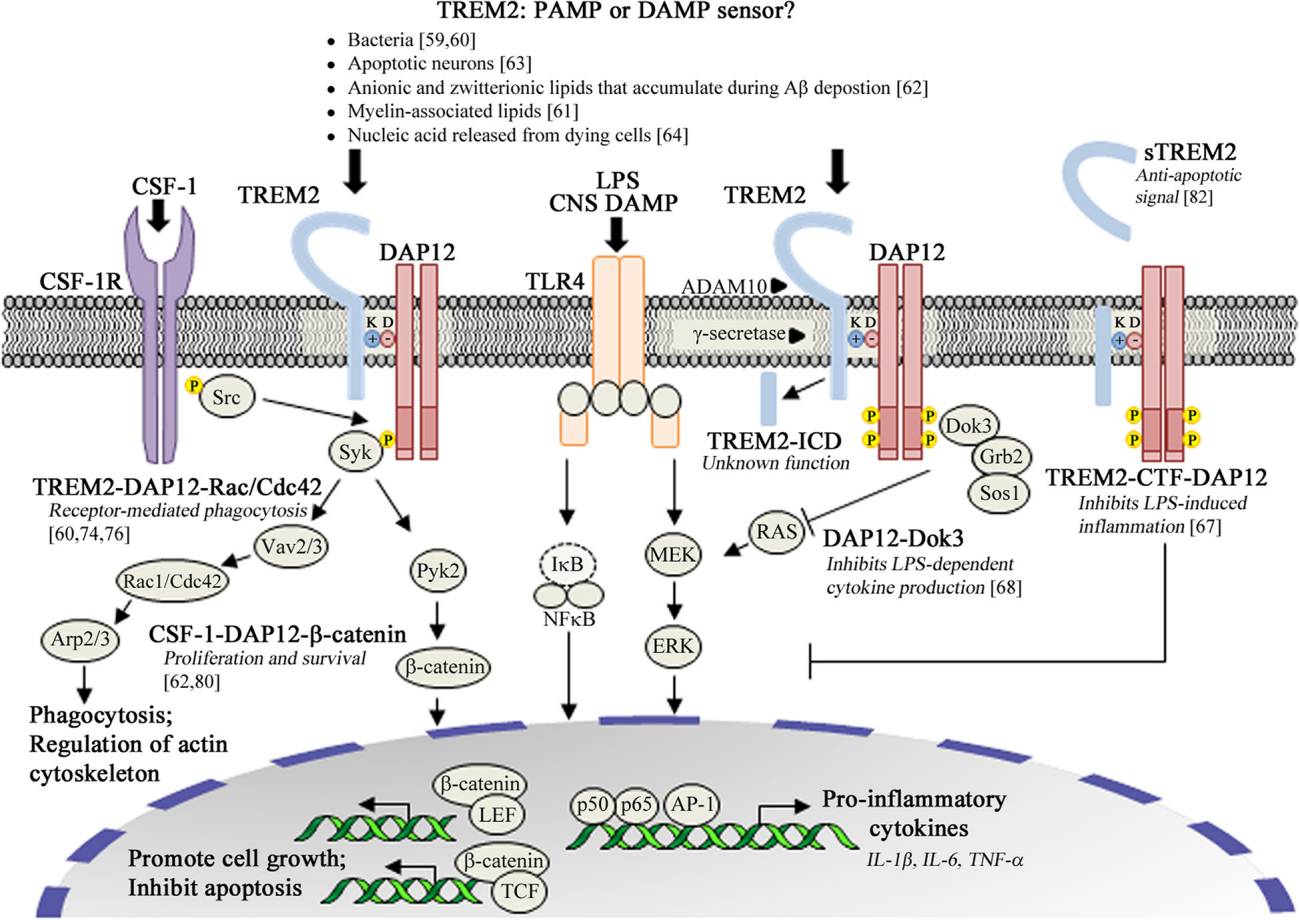
The TREM2-DAP12 axis modulates homeostatic functions via crosstalk with diverse signaling pathways. In the CNS, TREM2-DAP12 is preferentially expressed within microglia [32, 33]. Although signaling events need to be sequentially validated in microglia that endogenously express TREM2-DAP12, genetic depletion experiments *in vitro* support a multi-faceted role for TREM2-DAP12 in CNS homeostasis; *TREM2-DAP12 suppresses inflammatory cytokine production following PRR-mediated recognition of PAMPs/DAMPs.* LPS stimulation induces DAP12-dependent phosphorylation of Dok3 which is then recruited to the plasma membrane. There, pDok3 binds Grb2 and Sos1, thus preventing the activation of RAS and ERK, as well as the subsequent production of pro-inflammatory cytokines [68]. Similar to full-length TREM2, expression of only the TREM2-CTF leads to DAP12-dependent anti-inflammatory effects [67]. *TREM2-DAP12 facilitates phagocytosis of PAMPs/DAMPs and promotes cellular debris clearance following injury or insult.* Src kinase phosphorylation of the tyrosine residues of the DAP12 ITAM domain creates a docking site for Syk and downstream activation of the guanine nucleotide exchange factors Vav2/3 [74, 75] which are capable of activating Rac1/Cdc42-dependent phagocytosis and regulation of the actin cytoskeleton [60]. *TREM2-DAP12 signaling synergizes with that of CSF-1R to induce a transcriptional profile reflective of enhanced myeloid cell proliferation and reduced cell death*. CSF-1 via CSF-1R induces phosphorylation of DAP12 by Src kinases associated with tyrosine residue 559 of the CSF-1R, followed by DAP12-dependent phosphorylation of Syk and subsequent Pyk2-dependent activation of β-catenin-regulated pro-survival pathways [80, 81]

In an effort to investigate the molecular mechanism by which TREM2-DAP12 attenuates TLR4-mediated signaling, Peng and colleagues stimulated BMDMs from WT mice with LPS, and thereafter immunoprecipitated cell lysates with an anti-DAP12 antibody [68]. They found that the adaptor protein Dok3 was associated with the ITAM motif of DAP12 via the phosphotyrosine-binding domain. In a Src-dependent but Syk-independent manner, phosphorylated-Dok3 translocated from the cytoplasm to the plasma membrane where it associated with Grb2 and Sos1 which prevented activation of the RAS-ERK pathway (Fig. 1) [68]. Therefore, Dok3 participates in DAP12-mediated inhibition of TLR4-induced secretion of pro-inflammatory cytokines by blocking the ERK pathway. These results provide mechanistic insight into previous observation that *Dap12*^*−/−*^ BMDMs exhibit increased phosphorylated ERK in response to LPS stimulation [66].

Experiments using TLR DAMPs further support the hypothesis that TREM2-DAP12 is able to modulate pro-inflammatory signaling. In microglia, TREM2 or DAP12 knockdown led to significant increases in TNF-α, IL-1β, and IL-6 when incubated with apoptotic neurons [63] or Aβ [69]. However, contrary to these results, Jay et al. recently reported that in a mouse model of AD, TREM2 deficiency actually reduced pro-inflammatory cytokine levels (IL-1β and IL-6) in brains of APPPS1-*Trem2*^*−/−*^ mice [70]. One potential explanation for this discrepancy, the authors contend, is that the CNS-infiltrating monocyte-derived macrophage, as opposed to the resident microglia, is the key TREM2-expressing myeloid effector in AD pathogenesis [70]. Another explanation could be the timing in which inflammation was assessed—*in vitro,* inflammatory markers were assayed within hours to days of PAMP/DAMP stimulation [62, 63, 30, 65–69]; *in vivo*, the neuroinflammatory environment was assayed under conditions of constitutive PRR activation in brain lysates of four-month old mice [70]. In support of temporal cytokine fluctuations related to TREM2 signaling, a recent study measured IL-6 levels in peritoneal lavage fluid from WT and *Trem2*^*−/−*^ mice 6 and 20 h after LPS injection [71]. At 6 h, *Trem2*^−/−^ mice exhibited robustly increased IL-6 compared with controls. However, rapid resolution of the inflammatory response was observed in *Trem2*^*−/−*^ mice, leading to significantly decreased IL-6 levels at 20 h. Interestingly, when mice were challenged with *E. coli* in a pathogenic peritonitis model, at 16 h post-infection *Trem2*^−/−^ mice still maintained higher IL-6 levels in blood plasma compared with controls; yet, there was no statistically-significant survival advantage in either group. Since inflammation did not correlate with survival outcome, the authors suggested that TREM2 might concurrently affect the antimicrobial effector mechanism of bacterial phagocytosis, which was reduced both *in vitro* and *in vivo* in *Trem2*^−/−^ mice [71].

Overall, TREM2-DAP12 signaling likely modulates pro-inflammatory signals originating from TLR4 via intracellular DAP12-Dok3 pathway [68]. Additionally, as alluded to above, it is possible that production of TLR4-induced inflammatory cytokines could be blunted via indirect mechanisms; TREM2-mediated phagocytosis of pathogenic PAMPs and/or amyloidogenic DAMPs would deplete the pool of extracellular stimulatory ligands that, in excess, activate PRR-mediated production of pro-inflammatory cytokines.

## TREM2-DAP12 signaling facilitates phagocytosis

Shortly after the discoveries that linked genetic variants of DAP12 [9] and TREM2 [10] to the neurodegenerative disease NHD/PLOSL, Takahashi et al. sought out to investigate the role of TREM2-DAP12 within the CNS [63]. Specifically, they analyzed the vital microglial function of clearing apoptotic cells with or without intact TREM2 signaling. TREM2-shRNA or overexpression lentiviral vectors were used to transduce primary mouse microglia and then phagocytosis of apoptotic neurons was assayed *in vitro*. When TREM2 was knocked down, significantly fewer microglia phagocytosed apoptotic neuronal membranes compared with controls. Interestingly, when TREM2 was overexpressed the percentage of microglia that phagocytosed apoptotic neurons was increased which validated the role of TREM2 in phagocytosis [63]. Other experiments *in vitro* have yielded similar results; TREM2- or DAP12*-*deficient microglia incubated with Aβ [69, 72], *Trem2*^*−/−*^ peritoneal macrophages or microglia incubated with *E. coli* [71, 72]*,* and TREM2-depleted BV2 microglia incubated with apoptotic Neuro2A cells [73] showed a significantly attenuated capacity to phagocytose various PAMPs/DAMPs.

Importantly, N’Diaye et al. unequivocally demonstrated that TREM2-DAP12 expression is sufficient to facilitate phagocytosis *in vitro* [60]. Authors used the Chinese hamster ovary cell line that was transfected with a TREM2-DAP12 fusion construct and incubated with *E. coli*. Although these cells are typically considered to be non-phagocytic, they rapidly internalized bacteria in a TREM2-DAP12-dependent manner. Furthermore, this mechanism required Src kinase activity, tyrosine phosphorylation of the DAP12 ITAM domain, Syk, and the small guanosine triphosphates Rac1 and Cdc42 [60]. The Vav guanine nucleotide exchange factor family may also be a critical link in this TREM2-DAP12 signaling axis; Vav2 and Vav3 are mediators of DAP12-ITAM signals [74] and are capable of activating Rho-Rac/Cdc42 [75]. Although events need to be sequentially validated in myeloid cells that express endogenous TREM2, it is tempting to speculate that TREM2-DAP12 mediated phagocytosis can occur via Src-Syk-Vav2/3-Rac1/Cdc42-Arp2/3 (Fig. 1). Additional support for this model can be extrapolated from network analysis of critical TREM2-dependent signaling cascades in the human brain with significant enrichment for microglial-mediated cytoskeletal rearrangements necessary for phagocytosis—also highlighted within this network is the high degree of interconnectedness among TREM2, Vav, and Arp2/3 [76].

Data from *in vivo* experiments mirror *in vitro* findings and similarly support the ability of TREM2 to facilitate phagocytosis. In cell culture, when BMDMs were transduced with a TREM2*-*overexpression vector, phagocytosis of microsphere beads and apoptotic neurons was significantly increased [77]. In order to investigate whether this increased expression of TREM2 in peripheral myeloid cells could alter disease course in mice afflicted with the experimental autoimmune encephalomyelitis model of multiple sclerosis, myeloid cells were intravenously injected and cellular homing, myelin debris clearance, and disease progression were monitored. TREM2-transduced myeloid cells migrated to CNS lesions, facilitated myelin debris clearance and ameliorated clinical symptoms of disease. Intrigued by these findings, the authors suggested that TREM2 is an attractive target for promoting CNS repair in neuroinflammatory diseases [77]. Consistent with this hypothesis, TREM2 knockdown in BV2 microglia attenuated phagocytosis of oxygen-glucose-deprived neurons *in vitro*, and likewise TREM2 deficiency *in vivo* reduced phagocytosis of ischemic brain parenchyma in an experimental stroke model. Specifically, oil red O-positive staining was used to identify phagocytosed intracellular lipids within CNS tissue; approximately 50 positive cells per field were counted in WT ischemic mouse brains compared with <1 positive cell per field in *Trem2*^*−/−*^ mice. Importantly, brain infarct size was larger (less infarcted brain resorption) in *Trem2*^*−/−*^ mice which correlated with increased severity of neurological deficits [64].

Additionally, postmortem analyses of brain tissue from NHD/PLOSL patients revealed demyelinating lesions within the subcortical white matter [78]. In order to investigate how loss of TREM2 contributes to the pathogenesis of white matter tracts, cuprizone-induced nonautoimmune demyelination was assessed *in vivo* [61]. The cuprizone model is characterized by apoptosis of mature oligodendrocytes and activation of brain-resident microglia that remove damaged myelin. WT and *Trem2*^*−/−*^ mice were fed a cuprizone-containing diet for twelve weeks and then fed a cuprizone-free diet for two weeks and myelination was assessed by transmission electron microscopy. In WT mice, the corpus callosum contained remyelinated white matter tracks following the recovery phase. Conversely, corpus callosum in *Trem2*^*−/−*^ mice was largely demyelinated with visible deposition of myelin debris and axonal swelling. *In vitro,* purified lipid components such as sulfatide, sphingomyelin, and various phospholipids were capable of stimulating TREM2 signaling. Therefore, the authors proposed that microglia require TREM2 for myelin debris removal in a process that depends on TREM2 detection of lipid components exposed during myelin injury [61]. Contrary to these results, a recent paper presented evidence that TREM2 does not directly facilitate phagocytosis. Instead, it was proposed, TREM2 promotes microglial survival by synergizing with colony stimulating factor-1 receptor (CSF-1R) signaling [62].

## TREM2-DAP12 signaling promotes myeloid cell survival

Wang and colleagues observed that when *Trem2*^*−/−*^ mice were crossed to an AD mouse model, TREM2 deficiency neither had impact on microglial uptake of Aβ aggregates nor intracellular proteolytic processing of Aβ [62]. However, in brain sections from 5XFAD-*Trem2*^*−/−*^ mice there were more TUNEL-positive microglia compared with TREM2-expressing 5XFAD controls. Cell culture experiments using primary microglia expanded in conditioned media with colony-stimulating factor (CSF-1), which mimics the *in vivo* microenvironment [79], demonstrated that glia from 5XFAD-*Trem2*^*−/−*^ mice were significantly less viable than controls [62]. Earlier work by the same group provided valuable mechanistic insight—a CSF-1R-DAP12-β-catenin signaling network regulates survival and proliferation of myeloid cells [80]. *In vitro*, they showed that DAP12-deficient BMDMs had significantly impaired growth compared to WT cells when cultured for seven days with CSF-1. Specifically, a smaller percentage of DAP12-deficient BMDMs were in S and G2 phases compared with WT BMDMs [80]. Interestingly, retroviral-mediated DAP12 reconstitution restored cell-cycle profiles to approximately WT levels demonstrating that DAP12 is required for CSF-1-induced BMDM proliferation. Analysis of the molecular mechanism further revealed that CSF-1 via CSF-1R induces phosphorylation of DAP12 by Src kinases associated with tyrosine residue 559 of the CSF-1R, followed by DAP12-dependent phosphorylation of Syk and subsequent Pyk2-dependent activation of β-catenin (Fig. 1) [80, 81]. In this way, CSF-1 promotes proliferation and survival of myeloid cells via a DAP12-β-catenin-dependent pathway [80].

TREM2-DAP12-dependent signaling also prevents myeloid cell apoptosis during respiratory viral infection, albeit by a different molecular mechanism [82]. Cleavage of cell surface TREM2 to soluble TREM2 (sTREM2) has classically been speculated to result in formation of an inactive end product. However, Wu et al. recently presented data that counter conventional thought. The authors found that an increase in sTREM2 levels positively correlated with IL-13 production in diseased lung tissue and that exogenous administration of IL-13 (or IL-4 using the same receptor component) resulted in a time-dependent increase in sTREM2 released from BMDMs. Importantly, BMDMs treated with recombinant sTREM2 (amino acids 19–136) showed reduced cleaved caspase-3 and were spared from apoptosis (induced by CSF-1 withdrawal). This protective effect occurred in a sTREM2 dose-dependent manner, was irrespective of BMDM genotype (WT, *Trem2*^*−/−*^*,* or *Dap12*^*−/−*^), and was due to attenuated apoptotic signaling instead of cellular proliferation since BrdU incorporation was unaffected by sTREM2 treatment. The authors concluded that sTREM2 is unexpectedly active and capable of promoting macrophage survival [82]. Data that further support a neuroprotective role for sTREM2 is derived from analysis of cerebrospinal fluid samples from AD and FTD patients which shows significantly decreased levels of sTREM2 compared to healthy controls [72].

Since differentially cleaved forms of TREM2 are biologically active (including the plasma-membrane-bound TREM2-CTF [67] and the sTREM2 ectodomain [82]), insight into how TREM2 is transported to the cell surface and processed is necessary. Understanding the lifecycle of this receptor will determine whether, and how, these processes are affected by TREM2 mutations linked to neurodegenerative disease.

## TREM2 mutations linked to neurodegenerative disease—loss of function or gain of toxic function?

Most likely through glycosylation, membrane-bound TREM2 matures from a low-molecular weight immature form to a higher-molecular weight form ranging between 36 and 60 kDa. Once mature, TREM2 is shed from the membrane as 36–50 kDa sTREM2 by a disintegrin and metalloproteinase domain-containing protein, ADAM10 [72]. The membrane-bound CTF stub can still associate with and signal through DAP12 [67], but this fragment can also serve as a substrate for intramembrane-mediated γ-secretase cleavage [83] yielding an intracellular domain (ICD) with unknown function. Unlike non-mutated TREM2, expression of the TREM2 variants p.T66M and p.Y38C (associated with genetic risk of FTD [12, 16]) in HEK293 or BV2 microglial cells, led to accumulation of immature, membrane-bound TREM2 species (~36 kDa). Consistent with reduced maturation, p.T66M and p.Y38C mutants exhibited significantly diminished ADAM10-mediated proteolytic cleavage as evidenced by both decreased levels of sTREM2 and membrane-bound CTF [72]. The p.R47H mutant (associated with genetic risk of AD [13, 17–23], PD [14], ALS [24], and essential tremor [25]) showed slightly less, albeit similar patterns of cell-surface TREM2 expression, maturation, and sTREM2 generation compared with non-mutated TREM2 [72]. In order to assess the functional outcome of reduced mature, cell-surface TREM2 expression by the genetic mutants, a number of assays were used to investigate their phagocytic capacity. Interestingly, p.T66M and p.Y38C mutations significantly inhibited TREM2’s ability to mediate phagocytosis of latex beads, *E. coli*, and Aβ peptide. When the TREM2-R47H mutant was correspondingly used in assays, a decreased ability to phagocytose latex beads and Aβ peptide compared with TREM2 was observed. However, this effect was less pronounced in the p.R47H mutant compared with the other TREM2 variants, and p.R47H did not affect TREM2’s ability to mediate phagocytosis of *E. coli* [72]. In regards to TREM2-R47H, it appears that the largest effect was reduced levels of total TREM2 protein when transiently or stably expressed *in vitro* [72]. Therefore, it is possible that moderate effects on maturation and phagocytic capacity were solely driven by reduced TREM2 protein expression compared to control. Thus, in this context it is difficult to assess the functional nature of the p.R47H mutation.

Recently, another study investigated the biological effect of TREM2-R47H [62]. As previously discussed, Wang et al. found that TREM2 does not directly mediate phagocytosis, instead TREM2’s function in the CNS is two-fold—TREM2 is a sensor that detects lipids that are exposed during conditions of brain injury/insult and TREM2 promotes microglial survival via CSF-1R signaling [62]. Using the p.R47H variant, the authors reported that TREM2 cell-surface expression was unaffected by mutation. However, they found that the p.R47H mutation significantly reduced the ability of TREM2 to bind damage-associated lipids [62].

Overall, the p.T66M and p.Y38C mutations appear to alter trafficking and processing of TREM2 and impair TREM2-mediated phagocytosis of various ligands [72]. This effect of impaired phagocytic capacity mirrors *in vitro* findings using TREM2-deficient cell lines [63, 69, 71–73]. Therefore, these data support the hypothesis that TREM2 p.T66M and p.Y38C mutations exert loss-of-phagocytic function. However, results from p.R47H experiments are seemingly more ambiguous. If the p.R47H mutation encodes a TREM2 receptor that is unable to interact with microbial PAMPs and/or endogenous DAMPs, this would result in significant loss-of-biological function on multiple levels. Unlike TREM2 that supports receptor-ligand interaction, TREM2-R47H may have impaired ability to bind ligands that modulate homeostatic functions. However, it is also possible that the p.R47H mutation encodes a TREM2 receptor that no longer interacts with PAMPs/DAMPs and instead ectopically engages a novel ligand(s). Therefore, depending on the quantitative or temporal frequency at which this novel epitope is expressed, receptor-ligand interaction might result in aberrant signaling leading to a toxic-gain-of function. Similarly, TREM2-R47H may act as a decoy receptor by binding to a novel ligand and inhibiting engagement with its cognate receptor. In this way, TREM2-R47H could deplete the exogenous pool of a critical growth factor or survival signal, for example. To date, the biological consequence of mutations linked to neurodegenerative disease have only been evaluated in the context of TREM2 receptor trafficking and processing [72], phagocytosis [72], and DAMP sensing [62]. However, whether mutant TREM2-expressing microglia affect the inflammatory milieu of their CNS microenvironment or whether they participate in cell survival signaling has yet to be evaluated *in vitro* or *in vivo*.

## Conclusion

Microglia are an essential cadre of innate immune cells within the brain parenchyma entrusted with CNS defense against invading pathogens and the orchestration of tissue repair and regeneration following injury. Microglia are able to propagate diverse receptor signals and translate them into cell-type specific instructions for neurons and other glia through a delicate balance of pro- and anti-inflammatory paracrine signals. Therefore, it is understandable that mutations in receptors preferentially expressed on microglia within the CNS have been shown to increase the risk of developing neurological diseases [10–25, 1, 5–7]. TREM2 is a premier example of how mutations in a microglial receptor can lead to aberrant innate immune cell signaling that contributes to the initiation and propagation of neurodegenerative phenotypes including NHD/PLOSL [10, 11], FTD [12–17], AD [13, 17–23], PD [14], ALS [24], and essential tremor [25].

TREM2 and DAP12 genetic depletion studies *in vitro* and *in vivo* support three main roles for TREM2-DAP12 signaling (Fig. 1): (1) TREM2-DAP12 suppresses inflammatory cytokine production following PRR-mediated recognition of PAMPs/DAMPs; (2) TREM2-DAP12 facilitates phagocytosis of PAMPs/DAMPs and promotes cellular debris clearance following injury or insult; and (3) TREM2-DAP12 signaling induces a transcriptional profile reflective of enhanced myeloid cell proliferation and reduced cell death.

To date, numerous studies have investigated the genetic link between TREM2 variants and neurodegenerative diseases [10–25]; however only two studies have empirically tested the functional outcome of these mutations [62, 72]. TREM2 p.T66M and p.Y38C mutations appear to alter trafficking and processing of TREM2 and impair TREM2-mediated phagocytosis of numerous ligands [72] which supports the hypothesis that p.T66M and p.Y38C mutations exert loss-of-biological function, at least in the context of phagocytosis. If this indeed is the case, then TREM2-deficient cell lines and mice would serve as ideal platforms to test how loss of TREM2 impacts pathogenesis under conditions that simulate neurodegenerative disease.

Conversely, the biological significance of the p.R47H mutation is less clear. Wang et al. contend that the p.R47H mutation encodes a TREM2 receptor that is unable to interact with endogenous ligands [62]. Since TREM2 inhibits pro-inflammatory cytokine production, facilitates phagocytosis, and promotes cell survival, this finding could imply a significant loss of homeostatic function. However, it is also possible that TREM2-R47H is able to ectopically engage different epitopes and may therefore assume novel roles via aberrant signaling or sequestration of critical ligands. What is clear is that future work must focus on understanding the functional outcome of the p.R47H mutation given its genetic link to numerous neurodegenerative diseases [13, 14, 17–25]. *In vitro* and *in vivo* experiments utilizing TREM2-R47H-overexpression constructs should first determine how microglia expressing this variant are able to interact with other cells within their CNS microenvironment during homeostasis and normal aging. Once this relationship has been established, subsequent experiments should investigate the phenotype of TREM2-R47H-expressing microglia under conditions that mimic early steps in the neurodegenerative disease process (e.g., Aβ accumulation and amyloid plaque deposition in AD).

Lastly, it is important to consider, and empirically determine, whether the biological outcome of TREM2 mutation is, in fact, due to full-length, cell-surface, membrane-bound TREM2 receptor signaling, or whether detrimental effects may be mediated via altered production of other biologically-active TREM2 cleavage products, such as sTREM2, TREM2-CTF, or TREM2-ICD.
